# *Stigmaphyllon patricianum-firmenichianum* (Malpighiaceae), a new species from Loyalty Islands, New Caledonia

**DOI:** 10.3897/phytokeys.55.5472

**Published:** 2015-08-11

**Authors:** Jean-François Butaud

**Affiliations:** 1 Conservation International, 58 bis avenue de la Victoire, 98800 Nouméa, New Caledonia; 2 Consultant in forestry and Polynesian botany, P.O. Box 52832 - 98716 Pirae, Tahiti, French Polynesia

**Keywords:** *Stigmaphyllon*, *Ryssopterys*, Malpighiaceae, Loyalty Islands, New Caledonia

## Abstract

A new species of *Stigmaphyllon* (Malpighiaceae) is described: *Stigmaphyllon patricianum-firmenichianum* Butaud. It is restricted to the coral islands of Ouvéa, Lifou and Maré in the Loyalty Islands Province (New Caledonia) and is most similar to *Stigmaphyllon discolor* (Gand.) C.E.Anderson, known from New Caledonia and Solomon Islands. Previously, plants now known as *Stigmaphyllon patricianum-firmenichianum* were included in *Stigmaphyllon taomense* (Baker f.) C.E.Anderson, endemic to the northern part of Grande-Terre and Belep Islands (New Caledonia). A new circumscription of *Stigmaphyllon taomense* is proposed. The regional key for New Caledonian species of *Stigmaphyllon* is updated.

## Introduction

The family Malpighiaceae is represented by three native genera in New Caledonia: *Acridocarpus* Guill. & Perr. with a single endemic species, *Tristellateia* Thouars with one indigenous species, and *Stigmaphyllon* A.Juss. with eight indigenous species, of which five are endemic (Morat et al. 2012); a sixth endemic is added here.

*Stigmaphyllon* was recently revised by Anderson (2011, 1997) in both the Old and the New World. The Old World species, known from South-East Asia and the Western Pacific, traditionally had been assigned to the genus *Ryssopterys* A.Juss., which was found to be nested in *Stigmaphyllon* (Davis and Anderson 2010). Anderson (2011) recognized this group as Stigmaphyllon subg. Ryssopterys, comprising 21 species, of which ten were described as new.

Of the New Caledonian species of *Stigmaphyllon*, only one is known in the Loyalty Islands, which Anderson included in *Stigmaphyllon taomense* (Baker f.) C.E.Anderson, a species of Belep Islands and the northern part of Grande-Terre, the main island of the New Caledonian archipelago, ca. 200 km west of the Loyalty Islands (Anderson 2011; Baker 1921). Formerly, specimens from the Loyalty islands (Lifou or Maré) were labelled *Ryssopterys timoriensis* (DC.) A.Juss. (e.g., Schmid 1966, 1967), a synonym of *Stigmaphyllon timoriense* (DC.) C.E.Anderson; *Stigmaphyllon timoriense* is not known from New Caledonia (Anderson 2011).

Examination of living plants and herbarium specimens, and discussions with specialists of the genus *Stigmaphyllon* (C.E. Anderson, pers. comm. 2014) and New Caledonian flora (G. Gâteblé, pers. comm. 2014) revealed that the Loyalty Islands taxon differs from *Stigmaphyllon taomense*. It is here described as *Stigmaphyllon patricianum-firmenichianum*. Stigmaphyllon subg. Ryssopterys now includes 22 species, of which nine occur in New Caledonia.

## Systematics

### Stigmaphyllon patricianum-firmenichianum

Taxon classificationPlantaeMalpighialesMalpighiaceae

Butaud sp. nov.

urn:lsid:ipni.org:names:77149114-1

#### Type.

New Caledonia, Loyalty Islands, Ouvéa, Banutr, bord de route menant à l’aéroport, 20°38.345'S, 166°33.726'E, 11 m alt., liane de 5 m à fleurs mâles de couleur jaune, en lisière de forêt secondaire à *Podonephelium* et *Elattostachys*, 17 December 2013, *J.-F. Butaud 3346* (holotype NOU!, isotype P!).

#### Diagnosis.

*Stigmaphyllon patricianum-firmenichianum* is most similar to *Stigmaphyllon discolor* (Gand.) C.E.Anderson and *Stigmaphyllon mcphersonii* C.E.Anderson in the tomentose vesture of the abaxial surface of the blade which differentiates them from the other New Caledonia *Stigmaphyllon* species. *Stigmaphyllon patricianum-firmenichianum* differs from *Stigmaphyllon discolor* by the number of stamens, respectively 10 and 12–16, and by the number of flowers in each umbel, respectively 4–9 and 8–18(–20), and from *Stigmaphyllon mcphersonii* by the number of functional styles of the male flowers, respectively 3 and none (styles absent or rarely 1, rudimentary and without stigma), by the sepals length, respectively 2.8–3 mm and 1.5–2 mm, by the petals length, respectively 8–10 mm and 6–27 mm, and by the dorsal wing of samara, respectively 2.6–2.9 cm and 1.7–2 cm long.

#### Description.

Liana to over 8 m long; young stems tomentose, the vesture caducous in older parts, eventually becoming glabrate to glabrous. ***Blade*** of the larger leaves 4.8–7.5 × 3–5.4 cm, suborbicular to broadly ovate or ovate, apex emarginate to obtuse, acute or apiculate, base cordate to truncate, adaxially tomentose or sericeous when young, soon glabrescent to glabrous or with some hairs retained on costa, secondary veins and near the petiole, abaxially tomentose, eventually sericeous in older leaves, but in some leaves the vesture unevenly deciduous except close to the costa and at the apex and the petiole where always dense, secondary veins 4–7 pairs, prominent abaxially; marginal glands 0.1–0.3 mm diam.; petiole 1.3–2.6 cm long, tomentose, in older leaves the vesture sloughed off in patches, with a pair of glands borne at apex or partly on the base of the blade above insertion of the petiole, each gland 0.5–0.6 mm diam., slightly prominent; stipules 1 on each side of petiole, narrowly triangular, bractlike, to 1 mm long, abaxially tomentose, sometimes hidden by stem vesture. ***Hermaphrodite flowers*** 5–9 in each umbel or condensed pseudoraceme; umbels solitary or borne in dichasia; inflorescence stalks 1.1–2.9 cm long, often terminating a pair of foliaceous bracts, peduncles 3–5 mm long, pedicels 4–8 mm long, both tomentose; bracts c. 1 mm long, narrowly triangular, bracteoles c. 1 mm long, narrowly triangular, bracts and bracteoles abaxially tomentose. ***Sepals*** 5, 2.8–3 × 2.2–2.5 mm, orbicular or broadly ovate, abaxially densely sericeous but often glabrous along the margin. ***Petals*** 5, yellow, obovate with a claw 0.5–1 mm long, limb 8–10 × 5.5–7 mm, base acute or truncate, margin subentire or shallowly erose. ***Stamens*** 10; filaments c. 3 mm long; anthers without apiculum, glabrous. ***Ovary*** c. 1.6 mm long; 3 free styles c. 3.5 mm long, c. 0.1 mm diam., stigma c. 0.3 mm diam., peltate. ***Male flowers*** in inflorescences, and with sepals and petals, similar to hermaphrodite flowers: stamens 10, filaments c. 3 mm long; anthers without apiculum, glabrous; ovary rudimentary, a tiny mound of tissue embedded in a tuft of hairs; styles 3, c. 3 mm long, c. 0.1 mm diam., free or 2 variously united, stigma c. 0.3 mm diam., peltate. ***Fruit***: a schizocarp splitting into 3 samaras, pedicels 4–7 cm long. ***Dorsal wing of samara*** 2.6–2.9 × 1.1–1.4 cm; nut 4–5 mm long, c. 4 mm diam., broadly ovoid to spheroid, with prominent ridges, lateral winglets absent; areole 2.5–3 mm long and wide.

**Figure 1.**
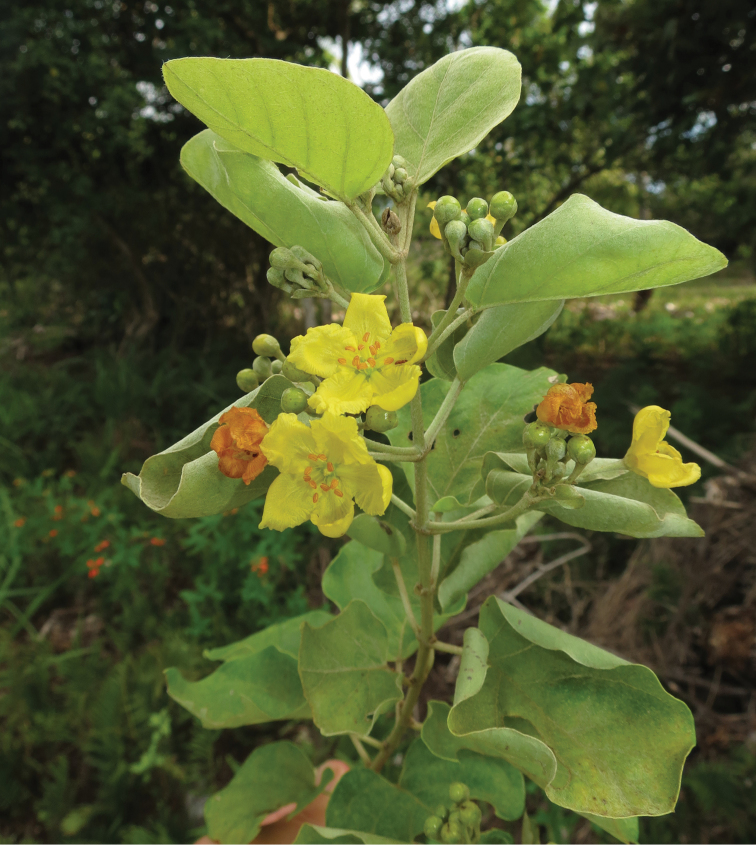
Male flowers of *Stigmaphyllon patricianum-firmenichianum* Butaud on Ouvéa atoll in December 2013 (specimen *Butaud 3346*).

**Figure 2.**
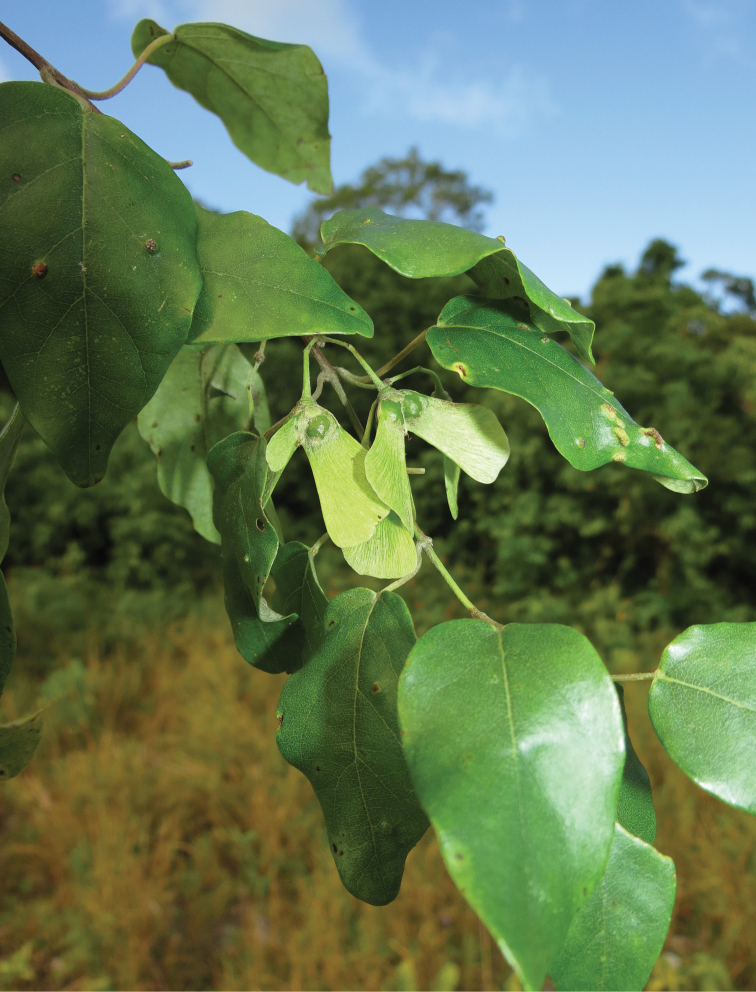
Fruits of *Stigmaphyllon patricianum-firmenichianum* Butaud on Lifou island in April 2015 (specimen *Butaud 3426*).

#### Phenology.

Flowers (emitting a pleasant odor) from November to April; fruits from February to May.

#### Distribution.

New Caledonia, endemic to Loyalty Islands; known only from Ouvéa, Lifou and Maré islands. Not recorded on the smaller islands of Beautemps-Beaupré, Tiga and Walpole.

#### Habitat.

This species is restricted to the calcareous soils of uplifted atolls between 5 and 90 m elevation, in littoral open forest or shrubland, and in the interior on plateau open forest or shrubland. It is also commonly found among the naturally open and shrubby vegetation of the cliffs between littoral and plateau. This vine is characteristic of fallows, young shrublands and secondary forests following shifting cultivation. It is also commonly found along roads and close to villages in open and sunny areas. The vegetation is often composed of trees and shrubs, such as *Acacia spirorbis* Labill., *Acronychia laevis* J.R.Forst. & G.Forst., *Elattostachys apetala* (Labill.) Radlk., *Glochidion billardierei* Baill., *Morinda citrifolia* L., Pipturus argenteus (G.Forst.) Wedd. var. lanosus Skottsb., *Podonephelium homei* (Seem.) Radlk., and Polyscias bracteata (RVig.) Lowry subsp. bracteata.

#### Conservation status.

Using the categories and criteria of IUCN (2001), the IUCN Red List Category Least Concern (LC) for *Stigmaphyllon patricianum-firmenichianum* is proposed. Indeed, this is a common vine of anthropized areas on the three islands, i.e. in villages, cultivated areas, fallows, shrubland, roadsides, and open littoral forest on the calcareous cliffs. No decline is estimated or has been documented.

#### Etymology.

I am pleased to name this new species for Patrick Firmenich (b. 1962), former Chief Executive Officer of Firmenich, a leading Swiss company creating fragrances and flavors. The Firmenich Charitable Foundation is supporting the sustainable management of the Loyalty Islands biodiversity, especially on Ouvéa atoll, a natural World Heritage site.

#### Common names.

The common names recorded for *Stigmaphyllon patricianum-firmenichianum* are “watoma” on Lifou (herbarium specimens *Deplanche 74*, *Däniker 2468* & *Bergeret 86*; Däniker 1932; Lenormand 1999, 1968) and “tai” on Maré (herbarium specimen *Däniker 2497*; Däniker 1932; Dubois 1971; Lormée et al. 2011). No common name has been recorded on Ouvéa.

#### Discussion.

*Stigmaphyllon patricianum-firmenichianum* is the sole member of the genus *Stigmaphyllon* in the Loyalty Islands. It is allied with *Stigmaphyllon discolor* and *Stigmaphyllon mcphersonii* with which it shares an abaxial tomentose vesture of the blade. Its inclusion under *Stigmaphyllon taomense*, a species with an abaxially sericeous blade, by Anderson (2011), may stem from the patchily deciduous vesture of some leaves, which can give the impression of a sericeous blade. Moreover, *Stigmaphyllon patricianum-firmenichianum*, *Stigmaphyllon mcphersonii* and *Stigmaphyllon taomense* have all 10 stamens, whereas *Stigmaphyllon discolor* bears 12–16 stamens. Nevertheless, its closest affinity in New Caledonia apparently is with *Stigmaphyllon discolor*, which occurs on most of the southern part of Grande-Terre and Isle of Pines. In South-East Asia and the Western Pacific, it is most similar to *Stigmaphyllon albidum* (Blume) C.E.Anderson, which can be differentiated by the absence of a style in male flowers. This new species is also clearly different from the widely distributed *Stigmaphyllon timoriense*, which has male flowers usually without styles and blades abaxially sericeous to glabrate.

#### Specimens examined.

Loyalty Islands. Lifou, *E. Deplanche 74* (P scan!); Lifou, Mou, 28 November 1925, *A.U. Däniker 2468* (Z [3] scan!); Lifou, 1927, *C. Bergeret 86* (P scan!); Lifou, We-Kodegni, forêt mi-dense, halliers, 14 February 1966, *M. Schmid 1038* (NOU!, P scan!, MICH n.v.); Lifou, Mutchaweng, 30 m, forêt sur terrain plat caillouteux, 18 February 1974, *H. MacKee 28179* (P scan!, MICH n.v.); Lifou, hauteurs au SE du Cap Lafon, 90 m, forêt saxicole, 20 February 1974, *H. MacKee 28295* (P scan!, MICH n.v.); Lifou, Wanaham, bord de route menant à Hnacaom, 20°47.037'S, 167°13.979'E, 38 m alt., liane de 5 m à fleurs femelles jaunes odorantes et jeunes fruits, en lisière de forêt secondaire à *Acacia*, *Glochidion*, *Polyscias*, *Acronychia*, *Secamone*, 15 April 2015, *J.-F. Butaud 3424* (NOU!, P!); Lifou, Xodre, bord de route menant au plateau, 21°7.682'S, 167°24.403'E, 58 m alt., liane de 5 m à fruits matures, en lisière de forêt de corniche à *Ficus virgata*, *Pipturus*, *Mucuna*, *Morinda*, 15 April 2015, *J.-F. Butaud 3426* (NOU!, P!); Maré, Tadine, 15 December 1925, *A.U. Däniker 2497* (Z [2] scan!).

### Circumscription of Stigmaphyllon taomense

Taxon classificationPlantaeMalpighialesMalpighiaceae

Stigmaphyllon taomense (Baker f.) C.E.Anderson, Blumea 56(2011)99 ≡
Ryssopterys taomensis Baker f., J. Linn. Soc., Bot. 45(1921)278.

#### Type.

New Caledonia, Mt Taom, 200 ft, 30 November 1914, *R.H. Compton 2286* (holotype BM scan!).

#### Discussion.

The description given by Anderson (2011) for *Stigmaphyllon taomense* is still correct, despite the separation of *Stigmaphyllon patricianum-firmenichianum* from it. The examination of *Stigmaphyllon taomense* specimens in NOU has shown some differences between Belep Islands and Northern Grande-Terre plants, especially the absence of marginal glands on the blade for the latter. Study of more specimens of both provenances may lead to the description of a new species endemic to Belep Islands.

#### Specimens examined.

New Caledonia, Grande Terre. Pain de Sucre, 22 December 1950, *A. Guillaumin* & *M.G. Baumann*-*Bodenheim 9734* (Z scan!, P scan!, BRI n.v.); Crêtes calcaires rocheuses au SE de la corne de Koumac, 250 m, 27 December 1972, *H. MacKee 26101* (P scan!, MICH n.v.); Koumac, 13 February 1969, *M. Schmid 2705* (NOU!, P scan!); Ile Art, plateau Nord, 150 m, 8 December 1975, *H. MacKee 30401* (NOU!, P scan!, MICH n.v.); Belep, 9 December 1975, *M. Debray 2466* (P scan!); Koumac, Oué Ambouch, 200 m, 24 January 1979, *H. MacKee 36515* (NOU!, P scan!, MICH n.v); Belep, December 1978, *D. Bourret 1873* (NOU!)

### Key to New Caledonian species of *Stigmaphyllon*

(adapted from Anderson 2011)

**Table:** 

1	Petiole flanked on each side by 2–3 stipules, to 2 cm long and leaflike	***Stigmaphyllon grandifolium***
–	Petiole flanked on each side by 1 triangular stipule, to 1.5 mm long and bractlike (never leafy)	**2**
2	Blades abaxially tomentose, the vesture patchily deciduous in some leaves but still dense at the base or the apex	**3**
–	Blades abaxially sericeous or glabrous on the entire surface	**5**
3	Umbels with 8–18(–20) flowers; stamens 12–16	***Stigmaphyllon discolor***
–	Umbels with 4–9 flowers; stamens 10	**4**
4	Sepals 2.8–3 mm long; petals 8–10 mm long; male flowers with 3 styles, all free or 2 united; dorsal wing of samara 2.6–2.9 cm long	***Stigmaphyllon patricianum-firmenichianum***
–	Sepals 1.5–2 mm long; petals 6–7 mm long; male flowers without functional styles (styles absent or rarely 1, rudimentary and without stigma); dorsal wing of samara 1.7–2 cm long	***Stigmaphyllon mcphersonii***
5	Blades abaxially sericeous, the vesture patchily deciduous in older leaves	***Stigmaphyllon taomense***
–	Blades abaxially glabrous or with some scattered hairs	**6**
6	Pedicels entirely glabrous or the basal 1/4 sericeous, red	***Stigmaphyllon gymnopodum***
–	Pedicels densely sericeous, green but the colour obscured by the vesture	**7**
7	Stamens 10; petals 6–7 mm diam.; male flowers without styles; blades 0.3–2.7 cm wide, linear to oblong to narrowly elliptical or narrowly lanceolate	***Stigmaphyllon angustifolium***
–	Stamens 12–18; petals 9–10 mm diam.; male flowers with styles; blades 2.5–6 cm wide, narrowly lanceolate to elliptical to broadly elliptical to ovate	***Stigmaphyllon mackeeanum***

## Supplementary Material

XML Treatment for Stigmaphyllon patricianum-firmenichianum

XML Treatment for Stigmaphyllon taomense
